# Assessment of Climate-Health Curricula at International Health Professions Schools

**DOI:** 10.1001/jamanetworkopen.2020.6609

**Published:** 2020-05-28

**Authors:** Brittany Shea, Kim Knowlton, Jeffrey Shaman

**Affiliations:** 1 Department of Environmental Health Sciences, Mailman School of Public Health, Columbia University, New York, New York; 2 The Natural Resources Defense Council, New York, New York

## Abstract

**Importance:**

Researchers have published surveys on health professionals' perceptions of the possible association between climate change and health (climate-health) and assessed climate-health or planetary health curricula in medical schools. However, curricula on climate-health are still lacking and gaps in knowledge persist.

**Objective:**

To understand the state of climate-health curricula among health professions institutions internationally.

**Design, Setting, and Participants:**

A survey of 160 institutional members of the Global Consortium on Climate and Health Education, which includes international health professions schools and programs, was conducted from August 3, 2017, to March 1, 2018. The survey, hosted by Columbia University Mailman School of Public Health, used an online survey tool for data collection.

**Main Outcomes and Measures:**

The survey assessed climate-health curricular offerings across health professions institutions internationally, including existing climate-health educational offerings, method of teaching climate-health education, whether institutions are considering adding climate-health education, whether institutions received a positive response to adding climate-health curricula and/or encountered challenges in adding curricula, and opportunities to advance climate-health education.

**Results:**

Overall response rate to the survey was 53%, with 84 of 160 institutional responses collected; 59 of the responses (70%) were from schools/programs of public health, health sciences, or health professions; 15 (18%) were from medicine; 9 (11%) were from nursing; and 1 (1%) was from another type of health profession institution. Among respondents, 53 (63%) institutions offer climate-health education, most commonly as part of a required core course (41 [76%]). Sixty-one of 82 respondents (74%) reported that climate-health offerings are under discussion to add, 42 of 59 respondents (71%) encountered some challenges trying to institute the curriculum, and most respondents have received a positive response to adding content, mainly from students (39 of 58 [67%]), faculty (35 of 58 [60%]), and administration (23 of 58 [40%]).

**Conclusions and Relevance:**

Current climate-health educational offerings appear to vary considerably among health professions institutions. Students, faculty, and administration are important groups to engage when instituting curricula, and awareness, support, and resources may be able to assist in this effort.

## Key Points

**Question:**

What is the state of curricula on the possible association between climate change and health (climate-health) among health professions institutions internationally?

**Findings:**

In this survey that included responses from 84 health professions institutions internationally, 63% reported offering education on climate-health, most commonly as part of a required core course. Adding the topic is under discussion at most institutions, with respondents reporting challenges in trying to institute the curriculum and receiving a positive response to adding the content.

**Meaning:**

The survey suggests that there is a range of educational offerings on climate-health, gaps in those offerings at many institutions, and challenges and opportunities for instituting or developing curricula.

## Teaser

This survey study describes responses from schools/programs for health professionals on inclusion of courses addressing the possible association between climate change and health.

## Introduction

Climate change appears to have both direct and indirect effects on human health, which may increase globally, and some populations may be affected more than others. Factors associated with health include heat-related illness and death as well as increased risk of vector-borne diseases, cardiovascular disease, and respiratory conditions from degraded air quality and threats to food and fresh water supplies.^1^ Global health leaders, such as physicians, nurses, and public health professionals, are among the professional groups that need to understand the complex interactions of climate and health along with current and emerging health challenges.^2^ To try and build a healthier, more secure future, health professionals should be trained to prevent, mitigate, and respond to the health consequences of climate change. However, curricula and educational programs on the topic of climate-health^3^ (defined as the health consequences from anthropogenic climate change) are still lacking and gaps in knowledge persist.

Several researchers have published climate-health education research projects and surveys. A survey conducted in 2018 and 2019 evaluated planetary health^4^ (defined as a field focused on characterizing the human health factors associated with human-based disruptions of the earth’s natural systems) teachings in all 17 Canadian medical schools; results showed variation in coverage of planetary health topics from “minimal to no teaching, to some lecture-, case-, or project-based teaching.”^5^ The researchers also identified barriers to integrating the education, such as limited time in the busy medical curricula, and provided recommendations for adding content. The Pontificia Universidad Javeriana in Colombia conducted a survey in 2017 to determine the state of climate-health education among the 59 medical schools in Colombia.^6^ The survey revealed that, of 47 respondents, the topic of climate-health is offered at 53% of the medical schools; however, the topic is not a priority in curricula, does not have set guidelines, and is not taught by qualified professionals. Another research project searched the Curriculum Inventory database supported by the Association of American Medical Colleges for specific terms related to climate change and found that none of the medical schools in the database reported explicit inclusion of climate change education in their curriculum.^7,8^

Other researchers have published surveys on health professionals’ perceptions of climate-health topics. One study involved interviews with public health and climate change professionals to understand barriers to and opportunities for advancing work on climate-health.^9^ Sarfaty and colleagues^10,11,12,13^ published the findings from several surveys that were conducted to determine physicians’ perceptions of, experience with, and opinions on health factors that may be associated with climate change. The surveys found that physicians observed that climate change may affect human health and supported responses to address it.

The Global Consortium on Climate and Health Education (GCCHE) surveyed its members to understand the current state of climate-health curricula among health professions institutions internationally. The survey describes present practices and is intended as an initial assessment with the aim of improving this educational content, addressing gaps in knowledge on the topic, and helping to chart future GCCHE progress. The present study aimed to examine how many health professions schools and programs currently have climate-health curricula, including planetary health curricula; the existing course and programmatic offerings; plans for adding curricula; and challenges and opportunities for instituting the curricula.

## Methods

The GCCHE at the Columbia University Mailman School of Public Health designed and developed the survey in conjunction with the broader GCCHE Coordinating Committee, a group of 12 (7 at the time the survey was developed) researchers and professionals in the climate-health field with appointments outside Columbia University who help guide Consortium efforts. The survey was deemed exempt by the Columbia University Institutional Review Board. The Columbia GCCHE team reviewed the American Association for Public Opinion Research (AAPOR) reporting guidelines for survey studies and made efforts to devise clear questions of appropriate length that did not lead to bias; the team also characterized nonresponders to check for the association between nonresponse bias and the validity of the findings.

The goal of this study was to assess the climate-health curricular offerings at health professions institutions by surveying the GCCHE membership. Member institutions are health professions institutions including schools and programs of public health, nursing, and medicine. The primary contact person from each GCCHE member institution served as survey respondent. The survey was conducted from August 3, 2017, to March 1, 2018, and was sent to the primary contact person at 160 GCCHE member institutions. Surveys were voluntary, self-administered online, and introduced by an email from GCCHE staff with brief background information on the study. It was indicated in the survey instrument that the results of the survey would be aggregated before anonymous results were reported more widely; participants did not receive financial compensation.

The survey included 23 questions (eAppendix in the Supplement). Four questions pertained to demographic characteristics of the institution and the individual completing the survey; the other 19 questions were about the climate-health educational activities at the school/program. Responses to 5 questions were required, including the respondent’s name, email address, name of school, whether they offer climate-health education, and whether discussion is under way to add any climate-health offerings. Responses to the remaining 18 questions were optional, of which some were conditional (based on answers to previous questions); thus, the number of responses differs for each question. Several questions allowed respondents to select all answers that apply. Key words (ie, climate-health and planetary health) were not defined within the survey.

Responses were categorized into 4 school/program types: (1) public health, health sciences, and health professions; (2) medicine; (3) nursing; and (4) other. Each response received 1 type of designation, which was selected based on the name of the school/program or the category that most closely fit the school/program. Each institutional response was given 1 designation: a school/program of public health, nursing, medicine, or other. However, a school might also include a program of another type that was not counted in this analysis. For example, a medical school might also include a program of public health; however, for the purposes of this study, the response was counted only as a medical school.

To address the possibility of nonresponse bias, a sample of nonrespondents was characterized to determine whether members were more likely to respond to the survey if their institution offered education on climate-health than if their institution did not offer education on climate-health. This informal assessment was made by looking at institutions’ webpages and/or online course catalogs to see if nonrespondents were less likely to offer climate-health curricula. To make this assessment, the Columbia GCCHE team first looked at whether differences existed between respondent institutions’ climate-health educational offerings listed online and how these institutions responded in the survey. For a sample of 15 respondents, their institutions’ webpages and/or online course catalogs were examined to determine whether information was available online to support their survey response of whether they offer climate-health curricula. Next, for a sample of 15 nonrespondents, the webpages and/or online course catalogs of these institutions were searched to determine whether these institutions appeared to offer climate-health education. These 2 samples were then compared for differences.

## Results

The overall response rate for the survey was 53%, with 84 of 160 institutional responses collected (Table 1). Fifty-nine of the 84 responses (70%) were from schools/programs of public health, health sciences, or health professions; 15 responses (18%) were from medicine; 9 responses (11%) were from nursing; and 1 response (1%) was from another type of health profession institution. Regarding climate-health education currently offered, the institutions of 53 of 84 respondents (63%) offer climate-health education and 31 institutions (37%) do not. Thirty-seven of the 53 programs (70%) that offer climate-health education were from schools/programs of public health, health sciences, or health professions; 12 (23%) were from medicine; and 4 (8%) were from nursing. Of the 50 institutions offering climate-health education that responded, 33 institutions (66%) assess students’ knowledge of climate-health and 17 institutions (34%) do not assess students’ knowledge.

**Table 1.** Climate-Health Educational Offerings and Evaluations^a^

Survey question	Institutions surveyed, No.	Response, No. (%)	
		Yes	No
Recruitment	160	84 (53)	76 (48)
Does your school offer climate-health education?	84	53 (63)	31 (37)
Does your school offer planetary health modules, courses, or programs?	51	17 (33)	34 (67)
Has your school received evaluations from students on their experience of and/or satisfaction with the climate-health teachings?	49	27 (55)	22 (45)
Does your school assess students’ climate-health knowledge?	50	33 (66)	17 (34)

^a^ Climate-health refers to possible association between climate change and health.

Members were asked to describe the climate-health education that their school offers (Table 2). Forty-one of 54 respondents (76%) indicated that their institution provides a climate-health session as part of a required core course; 24 institutions (44%) offer a climate-health stand-alone elective course; and 21 institutions (39%) offer a climate-health session as part of a nonrequired course. Fewer institutions offer a climate-health doctoral program (4 [7%]), climate-health postdoctoral positions (4 [7%]), a climate-health stand-alone required course(1 [2%]), and/or a climate-health master’s or certificate program (1 [2%]).

**Table 2.** Types of Climate-Health Educational Offerings^a^

Survey question	Response, No. (%)
What climate-health education does your school offer?^b^	54
Climate-health session as part of nonrequired course	21 (39)
Climate-health session as part of required core course	41 (76)
Climate-health stand-alone elective course	24 (44)
Climate-health stand-alone required course	1 (2)
Climate-health master’s or certificate program	1 (2)
Climate-health doctoral program	4 (7)
Climate-health postdoctoral positions	4 (7)

^a^ Climate-health refers to possible association between climate change and health.

^b^ Respondents were asked to select all responses that apply.

The second main research question addressed how climate-health education is being taught in member institutions including this content. Of schools that offer a stand-alone course on climate-health, most courses are 3 credits (18 of 29 [62%] responses), ranging from 0.25 to 6 credits. Members were asked how long their climate-health educational offerings have been in place. The average course age reported by 48 respondents was approximately 5 years, ranging from less than 1 year to 17 years. Twenty-seven respondents from 49 schools (55%) reported that their school had received evaluations from students on their experience and/or satisfaction with climate-health teachings, whereas 22 institutions (45%) had not received such evaluations.

Members described the main goals of their overall climate-health curriculum, and responses included the following:

- Improving understanding of the science and factors responsible for climate change;
- Increasing awareness of and literacy on climate-health and relevance to health professions practice;
- Improving knowledge of methods or tools to analyze health risks and climate information;
- Developing skills to identify vulnerable populations and strengthening understanding of climate-health–relevant policies, including mitigation of climate change and adaptation actions and health disparities;
- Instigating behavior change and advocacy for sustainability in health care; and
- Improving climate-health communication skills.

The third main research question aimed to determine whether institutions are discussing adding climate-health content and what their experience with adding the curricula has been, including if they received a positive response to adding climate-health curricula and/or challenges.

Of 82 respondents, 61 individuals (74%) reported that the addition of climate-health offerings is under discussion and 21 individuals (26%) responded that nothing is being considered (Table 3). Almost all institutions have received a positive response to adding climate-health curricula. Of 58 respondents, 39 individuals (67%) have received a positive response from students, 35 (60%) from faculty, 23 (40%) from administration. Fifteen respondents (26%) selected other, which included positive responses from funders, professional organizations in the community, climate change nonprofit organizations, and departments of health as well as that it is too early to know or is unknown.

**Table 3.** Challenges, Responses, and Future Plans for Climate-Health Education^a^

Question	Respondents, No.	Response, No. (%)								
Are any climate-health offerings under discussion to add?^b^	82	Session as part of nonrequired course, 32 (39)	Session as part of required core course, 32 (39)	Climate-health stand-alone elective course, 22 (27)	Climate-health stand-alone required course, 6 (7)	Climate-health master’s or certificate program, 9 (11)	Climate-health doctoral degrees, 3 (4)	Climate-health postdoctoral positions, 4 (5)	Nothing being considered, 21 (26)	NA
Have you received a positive response to adding climate-health curriculum?^b^	58	Yes, from students, 39 (67)	Yes, from faculty, 35 (60)	Yes, from administration, 23 (40)	No, have not received a positive response, 1 (2)	Other, 15 (26)	NA	NA	NA	NA
Have you encountered any challenges in trying to institute climate-health curriculum?^b^	59	Yes, lack of interest or demand from students, 5 (8)	Yes, administration or other skepticism about climate-health science, 4 (7)	Yes, lack of funding/time to support its development, 20 (34)	Yes, lack of available staff time to work on its development, 24 (41)	Yes, no available space in the core curriculum, 17 (29)	Yes, lack of teaching materials and staff expertise, 14 (24)	Yes, competing institutional priorities/politics, 18 (31)	No challenges, 17 (29)	Other, 8 (14)

Abbreviation: NA, not applicable.

^a^ Climate-health refers to possible association between climate change and health.

^b^ Respondents were asked to select all responses that apply.

Members were asked if they have encountered any challenges in trying to institute climate-health curricula. Forty-two of 59 respondents (71%) encountered some challenges trying to institute the curriculum. Of 59 respondents, 24 individuals (41%) experienced a lack of available staff time to work on development of curricula, 20 individuals (34%) reported a lack of funding and/or time to support development, 18 individuals (31%) reported competing institutional priorities and/or politics, 17 individuals (29%) reported that there is no available space in the core curriculum, 14 individuals (24%) reported a lack of teaching materials and staff expertise, 5 individuals (8%) described a lack of interest or demand from students, and 4 individuals (7%) reported administrative or other skepticism about climate-health science. Seventeen respondents (29%) reported that they had no challenges and 8 individuals (14%) selected other; these responses included the following:

- Issues were encountered early in the implementation, but they have successfully moved beyond them;
- Virtually impossible at master of public health level owing to full course load of students;
- Need updated teaching materials; and
- A few students stating that the faculty member was trying to push a “liberal agenda.”

The fourth main research question aimed to discover the opportunities to advance climate-health education. Specifically, members were asked what they have found helpful and whether they have any partnerships on climate-health.

When asked what they have found helpful in instituting or developing climate-health curricula, 47 of 59 respondents (80%) reported interest from students, 46 respondents (78%) described interest from faculty, 26 respondents (44%) reported interest from administration, 10 respondents (17%) indicated support from donors, 4 respondents (7%) reported support from institutional board members, and 8 respondents (14%) selected other (Table 4). Other responses included support from community organizations, political support, being a member of groups and alliances, national and international collaboration, support from the dean, and broader university interest.

**Table 4.** Opportunities in Climate-Health Education^a^

Question	Respondents, No.	Response, No. (%)					
What have you found helpful in instituting or developing climate-health curriculum?^b^	59	Interest from students, 47 (80)	Interest from faculty, 46 (78)	Interest from administration, 26 (44)	Support from board member, 4 (7)	Support from donor, 10 (17)	Other, 8 (14)
Does your school currently have any partnerships on climate change and human health?^b^	48	Yes, with another academic institution on training, 13 (27)	Yes, with another academic institution on research, 16 (33)	Yes, with a nonacademic institution, 19 (40)^c^	Yes, with a funder, 8 (17)	No, 17 (35)	NA

Abbreviation: NA, not applicable.

^a^ Climate-health refers to possible association between climate change and health.

^b^ Respondents were asked to select all responses that apply.

^c^ For example, business, government, nongovernmental organization.

When we assessed the characteristics of survey respondents and nonrespondents, some evidence of bias was found, indicating that respondents were more likely to already offer climate-health curricula. Specifically, in the sample of 15 responding institutions, 9 institutions (60%) had indicated in the survey that they offer climate-health curricula. When searching these same institutions’ webpages and/or online course catalogs, 12 of 15 institutions (80%) appeared to either offer (n = 8) or probably offer (n = 4) climate-health curricula. Thus, there was a 20 percentage-point difference between information online and survey responses. From the sample of 15 nonresponding institutions, 10 institutions (67%) appeared to offer (n = 5) or probably offer (n = 5) climate-health curricula. These findings identify a slight difference in rates of offering climate-health curricula between respondents and nonrespondents to our survey.

## Discussion

This survey provides a view of the state of climate-health education at health professions schools and programs internationally. The survey results suggest that several institutions included in the survey have successfully integrated climate-health content into their curricula. Typically, of institutions in this survey, climate-health education is offered as part of a required core course (76%), as part of a nonrequired course (39%), or as a climate-health stand-alone elective course (44%). The breadth of educational offerings, number of credits offered, years since inception, and whether members conduct evaluations and assess learning reveal considerable variation among institutions. Many schools have offered climate-health curricula for several years, and most schools assess students’ climate-health knowledge (Table 5).

**Table 5.** Climate-Health Teaching Methods and Assessment^a^

Survey question	Response, No. (%)
If your school offers a stand-alone course on climate-health, what teaching methods are used?^b^	28
Laboratories	1 (4)
Lectures	24 (86)
In-class exercises	20 (71)
Online tutorials or MOOCs	8 (29)
Internships outside the classroom	2 (7)
How is climate-health knowledge assessed?^b^	34
Quizzes	19 (56)
Examinations	22 (65)
Papers	19 (56)
Capstone	5 (15)
Thesis	7 (21)
Dissertation	8 (24)

Abbreviation: MOOCs, massive open online courses.

^a^ Climate-health refers to possible association between climate change and health.

^b^ Respondents were asked to select all responses that apply.

Members were asked if they are discussing adding curricula, and results showed that most are discussing a session, whether part of a nonrequired course (39%) or required core course (39%), followed by a climate-health stand-alone elective course (27%). About a quarter of respondents are not considering further content (26%). The findings suggest that although most institutions are discussing adding curricula, they are considering adding content as sessions or elective courses instead of more substantial offerings or required courses.

Most respondents have encountered challenges in trying to institute climate-health curricula, and the 2 most cited issues are lack of available staff time (41%) and funding/time to support its development (34%), followed by competing institutional priorities/politics (31%). This finding suggests that challenges relate to available resources and institutional support, rather than demand for or interest in the content; 29% of respondents reported that they encountered the challenge of no available space in the core curriculum, and 24% reported lack of teaching materials and staff expertise. These data suggest that time and resource constraints as well as support for the content present challenges for instituting the curricula. There may be connections among the various challenges encountered by individual institutions. For example, the broader political context within a country might influence the priorities of an institution and present challenges for developing curricula. In addition, lack of funding could lead to an inability to hire faculty/staff with expertise in climate-health topics and insufficient time to work on the development of content, teaching materials, or training of staff.

Most respondents have received a positive response to adding climate-health curricula, and the positive response was principally from students, followed by faculty; only 40% of the respondents have received a positive response from administration. In addition, most respondents reported that interest of students, followed by interest of faculty, was helpful in instituting or developing climate-health curricula, and 44% responded that interest from administration was helpful. These findings suggest that students and faculty are important groups to work with and engage when planning to institute or develop curricula, and that interest from administration also appears to be important. Most respondents have a partnership on climate-health, and most of the partnerships are with another academic institution on research or with a nonacademic institution.

On the basis of these findings, providing resources to help institutions incorporate climate-health education into curricula, including information on how to integrate the curriculum in a way that synthesizes climate-health into existing course material, might help to accelerate the number of health professions schools and programs that offer this education. In addition, support for the curricula, in terms of both time and resources, and building off of the response and interest of students and faculty might help to institute curricula at individual institutions.

Climate-health resources from various groups around the world are available, both on the topic generally and specifically related to curricular development. Certain groups focus on climate-health, while others cover environmental health or planetary health topics more broadly. In addition, some resources are discipline specific, such as those developed for medical professionals, while others are tailored for all health professionals or relevant across all disciplines. The GCCHE provides a set of core climate-health competencies for all health professions students and a knowledge bank of resources and recommendations to help institutions integrate content into curricula.^14^ Curricular resources are also offered by organizations and groups, such as the National Institute of Environmental Health Sciences in the United States,^15^ which develops lesson plans, slides, and case studies on climate-health for clinical health care and public health students; the Alliance of Nurses for Healthy Environments, which offers curricular resources to help integrate environmental health topics into nursing curricula^16^; the NurSus project, which provides educational materials on sustainability topics for nurses in several languages^17^; and the World Organization of Family Doctors Working Party on the Environment, which develops curricula and shares educational materials on environmental health for family physicians.^18^ Schools and institutions are setting precedents by creating climate-health centers,^19,20,21,22^ certificates,^23^ curricular infusion projects,^24^ and fellowships.^25^ Health professions associations are taking a stance on climate change, and some have developed policies and motions on climate-health education,^26,27^ and students are organizing and taking the lead.^28,29,30^ Several articles have described why climate change education may be important in health professions curricula and how to integrate content without imposing a large time commitment.^8,24^ Ready-to-use and practical resources are also available to help educate and organize health professionals,^31,32,33^ and many organizations communicate information on climate-health in easily understood and relevant ways.^34,35^ All of these efforts suggest the growing importance of understanding, preparing for, and helping to prevent changes in human health that may be associated with climate change.

### Limitations

This study has limitations. The first is a response rate of 53%. The second is that participation was voluntary and from an overall self-selected group that does not include all health professions schools, which prevents assurance that the sample is truly representative of the overall state of climate-health education at all health professions schools internationally. A third possible limitation is the nonresponse bias identified above. Although this sample was small and did not show strong bias, potential for some bias in the overall sample is acknowledged.

## Conclusions

Climate change may be affecting health in a variety of ways with increasing consequences. Health professionals, including those in public health, nursing, and medical services, should be educated on how to prevent, mitigate, and respond to factors associated with climate change that may be associated with health in a negative way. We believe that the results of this survey may provide a baseline assessment of the current state of climate-health education internationally among health professions schools and programs.

The survey suggests that a range of educational offerings exist on climate-health, including sessions, courses, programs, and postdoctoral positions. Some schools have offered climate-health education for several years, some are just now adding content, and others do not include any content on the subject. Although many schools are discussing adding climate-health educational offerings, considerable gaps in offerings remain at several institutions as well as challenges that extend beyond the institutional level, such as political and funding priorities that might lead to lack of staff time and materials to support the training.

Opportunities also exist to facilitate the integration of curricula, such as working with students, faculty, and members of administrations who are interested in this topic. We suggest that health professions schools include this content in their curricula and that awareness as well as financial support, resources, and expertise increase to help in its uptake. To facilitate this integration, institutions can look to online resources, groups, and networks to provide guidance and information to develop curricula.

## Notes

Supplement. **eAppendix.** Survey Instrument
