# Supplementary material for: Assessment of Climate-Health Curricula at International Health Professions Schools
Source: JAMA Netw Open. 2020 May 28;3(5):e206609. doi: 10.1001/jamanetworkopen.2020.6609 (PMC7256668; doi:10.1001/jamanetworkopen.2020.6609)
Supplement: Supplement. — eAppendix. Survey Instrument [file jamanetwopen-3-e206609-s001.pdf]

## Supplementary Online Content

Shea B, Knowlton K, Shaman J. Assessment of climate-health curricula at international health professions schools. *JAMA Netw Open*. 2020;3(5):e206609. doi:10.1001/jamanetworkopen.2020.6609

**eAppendix.** Survey Instrument

This supplementary material has been provided by the authors to give readers additional information about their work.

© 2020 Shea B et al. *JAMA Network Open.*

**eAppendix. eSurveyInstrument**

COLUMBIA UNIVERSITY | MAILMAN SCHOOL of PUBLIC HEALTH   GLOBAL CONSORTIUM ON CLIMATE AND HEALTH EDUCATION

# Global Consortium on Climate and Health Education: Survey on Climate-Health Educational Efforts Internationally

**The Global Consortium on Climate & Health Education (GCCHE) aims to develop a global community among health professions schools interested in establishing or expanding climate-health education. The GCCHE is being led by the Columbia University Mailman School of Public Health, home to the US' first academic Climate and Health Program, with financial support from the Rockefeller Foundation. In order to establish a baseline for charting future progress, we are conducting this survey on the current state of climate-health curricula internationally among Consortium members.**

**As a GCCHE member, we would like to ask you to please complete the survey. If you have any questions or additional comments, please email the GCCHE Project Director, Brittany Shea (bes2161@cumc.columbia.edu). We appreciate your willingness to share this information, the results of which will be aggregated before anonymous results are reported more widely.**

***Thank you again for your participation in our baseline survey, and for your membership in the GCCHE***

* 1. Name

[ ]

2. Title

[ ]

* 3. Email address

[ ]

* 4. Name of your school

[ ]

* 5. Does your school offer climate-health education?

○ Yes

○ No (If "No," you will automatically skip to a question about whether you plan to offer some form of climate-health education)

© 2020 Shea B et al. *JAMA Network Open.*

## Global Consortium on Climate and Health Education: Survey on Climate-Health Educational Efforts Internationally

6. What climate-health education does your school offer? (Please select all that apply)

- ☐ Climate-health session as part of non-required course
- ☐ Climate-health session as part of required core course
- ☐ Climate-health standalone elective course (If selected, please answer Questions 7 and 8)
- ☐ Climate-health standalone required course (If selected, please answer Questions 7 and 8)
- ☐ Climate-health masters or certificate program (If selected, please answer Questions 9 and 10)
- ☐ Climate-health doctoral program
- ☐ Climate-health post-doctoral positions

COLUMBIA UNIVERSITY | MAILMAN SCHOOL of PUBLIC HEALTH GLOBAL CONSORTIUM ON CLIMATE AND HEALTH EDUCATION

## Global Consortium on Climate and Health Education: Survey on Climate-Health Educational Efforts Internationally

7. If your school offers a standalone course on climate-health, what is its title, and how many credits are received upon completion?

**Title:** ______________________

**Credits:** ______________________

© 2020 Shea B et al. *JAMA Network Open.*

8. If your school offers a standalone course on climate-health, what teaching methods are used? (Please select all that apply)

- [ ] Labs
- [ ] Lectures
- [ ] In-class exercises
- [ ] Online tutorials or MOOCs (Massive Open Online Courses)
- [ ] Internships outside the classroom



9. If your school offers a masters or certificate program in climate-health, what is its title, and how many credits are needed for its completion?

**Title:** ____________________

**Credits:** ____________________

10. If your school offers a masters or certificate program in climate-health, what teaching methods are used? (Please select all that apply)

- [ ] Labs
- [ ] Lectures
- [ ] In-class exercises
- [ ] Online tutorials or MOOCs (Massive Open Online Courses)
- [ ] Internships outside the classroom

© 2020 Shea B et al. *JAMA Network Open.*

## Global Consortium on Climate and Health Education:
## Survey on Climate-Health Educational Efforts Internationally

11. What are the main goals of your overall climate-health curriculum?

12. How long have the climate-health education teachings been in place?

13. Has your school received evaluations from students on their experience of and/or satisfaction with the climate-health teachings?

- ◯ Yes (If you are willing to share the results of the evaluations, please email Brittany Shea at bes2161@cumc.columbia.edu)
- ◯ No

COLUMBIA UNIVERSITY | MAILMAN SCHOOL of PUBLIC HEALTH GLOBAL CONSORTIUM ON CLIMATE AND HEALTH EDUCATION

## Global Consortium on Climate and Health Education:
## Survey on Climate-Health Educational Efforts Internationally

14. Does your school assess students' climate-health knowledge?

- ◯ Yes
- ◯ No

15. If "Yes," how is climate-health knowledge assessed? (Please select all that apply)

- ☐ Quizzes
- ☐ Exams
- ☐ Papers
- ☐ Capstone
- ☐ Thesis
- ☐ Dissertation

© 2020 Shea B et al. *JAMA Network Open.*

## Global Consortium on Climate and Health Education: Survey on Climate-Health Educational Efforts Internationally

16. If available, please provide the URL to your climate-health curriculum website:

17. Does your school offer Planetary Health modules, courses, or programs?

- ○ Yes
- ○ No

© 2020 Shea B et al. *JAMA Network Open.*

**Global Consortium on Climate and Health Education:**
**Survey on Climate-Health Educational Efforts Internationally**

* 18. Are your Planetary Health and climate-health modules or programs linked or integrated?

- ○ Yes
- ○ No

COLUMBIA UNIVERSITY | MAILMAN SCHOOL of PUBLIC HEALTH GLOBAL CONSORTIUM ON CLIMATE AND HEALTH EDUCATION

**Global Consortium on Climate and Health Education:**
**Survey on Climate-Health Educational Efforts Internationally**

19. Does your school currently have any partnerships on climate change and human health? (Please select all that apply)

- ☐ Yes, with another academic institution on training
- ☐ Yes, with another academic institution on research
- ☐ Yes, with a non-academic institution (business, government, NGO, etc.)
- ☐ Yes, with a funder
- ☐ No

© 2020 Shea B et al. *JAMA Network Open.*

* 20. Are any climate-health offerings under discussion to add? (Please select all that apply)

- [ ] Session as part of non-required course
- [ ] Session as part of required core course
- [ ] Climate-health standalone elective course
- [ ] Climate-health standalone required course
- [ ] Climate-health masters or certificate program
- [ ] Climate-health doctoral degrees
- [ ] Climate-health post-doctoral positions
- [ ] Nothing being considered

COLUMBIA UNIVERSITY | MAILMAN SCHOOL of PUBLIC HEALTH GLOBAL CONSORTIUM ON CLIMATE AND HEALTH EDUCATION

## Global Consortium on Climate and Health Education: Survey on Climate-Health Educational Efforts Internationally

21. Have you received a positive response to adding climate-health curriculum? (Please select all that apply)

- [ ] Yes, from students
- [ ] Yes, from faculty
- [ ] Yes, from administration
- [ ] No, have not received a positive response
- [ ] Other (please specify)

© 2020 Shea B et al. *JAMA Network Open.*

22. Have you encountered any challenges in trying to institute climate-health curriculum? (Please select all that apply)

- [ ] Yes, lack of interest or demand from students
- [ ] Yes, administration or other skepticism about climate-health science
- [ ] Yes, lack of funding/time to support its development
- [ ] Yes, lack of available staff time to work on its development
- [ ] Yes, no available space in the core curriculum
- [ ] Yes, lack of teaching materials and staff expertise
- [ ] Yes, competing institutional priorities/politics
- [ ] No challenges
- [ ] Other (please specify)

23. What have you found helpful in instituting or developing climate-health curriculum? (Please select all that apply)

- [ ] Interest from students
- [ ] Interest from faculty
- [ ] Interest from administration
- [ ] Support from Board members
- [ ] Support from donor
- [ ] Other (please specify)

© 2020 Shea B et al. *JAMA Network Open.*
